# Supplementary material for: Analysis of PPARγ Signaling Activity in Psoriasis
Source: Int J Mol Sci. 2021 Aug 10;22(16):8603. doi: 10.3390/ijms22168603 (PMC8395241; doi:10.3390/ijms22168603)
Supplement: Supplementary file 1 [file ijms-22-08603-s001.zip › Supplemental materials_Analysis of PPARg signaling activity in psoriasis/Pathway models/Models images and html files/PPARG inhibits phenotypes assotiated with psoriasis/124606.html]

edema


# Disease edema

|  |  |
| --- | --- |
| URN | urn:agi-meshdis:Edema |
| Total Entities | 41 |
| Connectivity | 6910 |
| Name | edema |

---

|  |  |
| --- | --- |
| ChildConcepts | brain edema |
|  | papilledema |
|  | posterior reversible encephalopathy syndrome |
|  | ankle edema |
|  | application site edema |
|  | arm edema |
|  | bone marrow edema |
|  | eye edema |
|  | eyelid edema |
|  | facial edema |
|  | foot edema |
|  | generalized edema |
|  | genital edema |
|  | hand edema |
|  | idiopathic edema |
|  | injection site edema |
|  | leg edema |
|  | intestinal edema |
|  | oral edema |
|  | noncardiogenic pulmonary edema |
|  | nasal edema |
|  | orbital edema |
|  | paw edema |
|  | periorbital edema |
|  | peripheral edema |
|  | postoperative edema |
|  | cutaneous edema |
|  | retinal edema |
|  | tongue edema |
|  | ear edema |
|  | chemosis |
|  | lymphedema |
|  | angioedema |
|  | cardiac edema |
|  | laryngeal edema |
|  | myxedema |
|  | pulmonary edema |
|  | corneal edema |
|  | anasarca |
|  | pharyngeal edema |
|  | lipedema |

---

|  |  |
| --- | --- |
| Pathway | PPARG inhibits phenotypes assotiated with psoriasis |

---

|  |  |
| --- | --- |
| MedScan ID | 9010810 |

---

|  |  |
| --- | --- |
| Alias | venous edema |
|  | Hydrops |
|  | Interstitial Edema |
|  | adjuvant edema |
|  | hydropsy |
|  | reactive edema |
|  | edemas |
|  | Dropsy |
|  | pitting edema |
|  | Interstitial Edemas |
|  | non pitting edema |
|  | edema |
|  | nonpitting edema |
|  | dropsies |

---

|  |  |
| --- | --- |
| MeSH ID | D004487 |
|  | M0007051 |

---

|  |  |
| --- | --- |
| MeSH Heading | Edema |

---
